# Real-time Measurement of Biomagnetic Vector Fields in Functional Syncytium Using Amorphous Metal

**DOI:** 10.1038/srep08837

**Published:** 2015-03-06

**Authors:** Shinsuke Nakayama, Tusyoshi Uchiyama

**Affiliations:** 1 Department of Cell Physiology, Nagoya University Graduate School of Medicine, Nagoya 466-8550, Japan; 2 Department of Electronics, Nagoya University of Graduate School of Engineering, Nagoya 464-8603, Japan

## Abstract

Magnetic field detection of biological electric activities would provide a non-invasive and aseptic estimate of the functional state of cellular organization, namely a syncytium constructed with cell-to-cell electric coupling. In this study, we investigated the properties of biomagnetic waves which occur spontaneously in gut musculature as a typical functional syncytium, by applying an amorphous metal-based gradio-magneto sensor operated at ambient temperature without a magnetic shield. The performance of differentiation was improved by using a single amorphous wire with a pair of transducer coils. Biomagnetic waves of up to several nT were recorded ~1 mm below the sample in a real-time manner. Tetraethyl ammonium (TEA) facilitated magnetic waves reflected electric activity in smooth muscle. The direction of magnetic waves altered depending on the relative angle of the muscle layer and magneto sensor, indicating the existence of propagating intercellular currents. The magnitude of magnetic waves rapidly decreased to ~30% by the initial and subsequent 1 mm separations between sample and sensor. The large distance effect was attributed to the feature of bioelectric circuits constructed by two reverse currents separated by a small distance. This study provides a method for detecting characteristic features of biomagnetic fields arising from a syncytial current.

Many tissues have a cellular organization designed to conduct an electric current, so as to achieve their functions. Nerve impulses are conducted in axonal fibers, conveying cellular information toward target cells. Cardiac pacemaker potentials propagate throughout the atrium and ventricle to produce synchronized heart beats. Other examples of functional syncyti (i.e. many cells electrically coupled to act synchronously) exist throughout the body, especially in the autonomic nervous system. The well-known coordinated motions of gut musculature, such as peristalsis and segmentation^1^,^2^, are one particular instance of cooperative electric activities of the syncytium.

The conduction of an electric current induces a magnetic field. This should hold true for biological systems. Magnetometers to measure biomagnetic fields would thus provide non-invasive and aseptic estimations of how cellular organizations electrically communicate and affect function. Such devices would need to be sensitive to detect small signals from small biological samples, ideally in real time and should not an need elaborate or expensive infrastructure, so that they can be routinely and universally used in laboratory and hospital settings. Current methodologies to detect biomagnetic fields are operated with several requirements. For instance, superconducting quantum interference devices (SQUID) are placed in a liquid coolant container and thus limit their ease of use^3^,^4^. In the case of atomic magnetometers, significant heating (180–200°C) is normally applied to produce sufficient alkaline metal vapors for the necessary sensitivity^5^. Also, both magnetometers need to be shielded against the geomagnetic field, because of saturation.

In this study, we show quasi-real-time measurements of biomagnetic vector fields in typical functional syncytia of gut musculatures, by using an improved amorphous metal-based magneto sensor, which is operated at ambient temperature without a magnetic shield. We incorporated a gradio-type magneto sensor device constructed with a single magnetic amorphous wire and a pair of transducer coils on both ends. In gut musculature samples isolated from guinea-pigs, magnetic waves up to several nT were stably observed under physiological conditions. The polarity of magnetic waves was altered depending on the relative angle of the muscle layer and magneto sensor, indicating the existence of propagating intercellular currents. We also observed a rapid reduction of the magnitude of biomagnetic fields within a small distance from the tissue. Our practical and computational simulations demonstrate that this can be attributed to the feature of bioelectric circuits constructed by a propagating intercellular current and extracellular return currents separated by a small distance.

## Results

### Magneto sensor system

**Figure 1:**
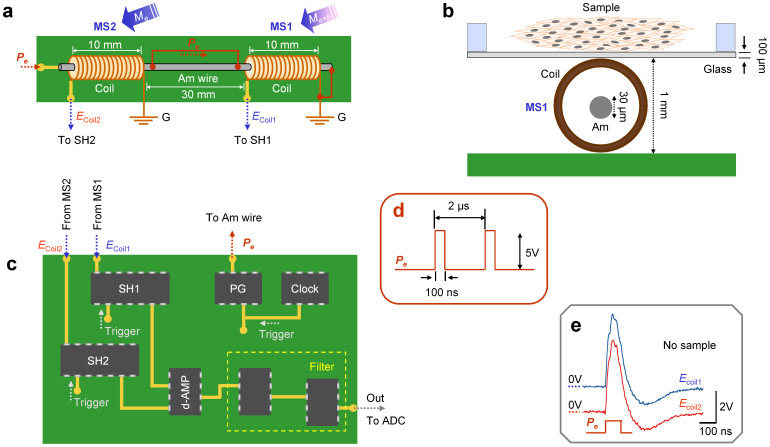
Schematic diagram of a gradio-magneto sensor system. (a) A gradio-magneto sensor device was composed of a single amorphous metal (Am) wire (50 mm in length) with a pair of detector coils (10 mm in length; 300 turns) mounted at both ends. MS1 was placed below a recording chamber, while MS2 was placed ~30 mm apart from MS1 in the same direction. MS1 and MS2 received driving electric pulses (*P_e_*). The intermediate part of the wire (30 mm) was electrically shunted. (b) The Am wire was placed in a plastic bobbin (~1 mm diameter) surrounded by a transducer coil. The sample was separated by a cover glass (100 μm thick). (c) A pulse gate IC (PG) triggered by a clock IC. The same clock IC also triggers sample-and-hold detectors (SH1, SH2) to measure the voltage of the transducer coils in MS1 and MS2. A fast operation amplifier (d-AMP) differentiates the voltage in SH2 from that in SH1. Output signals were filtered by high and low-cut filters (H/LPF: 0.5 Hz and 20 Hz) and stored in computer memory via an analog-to-digital converter (ADC). (d) *P_e_* (100 ns, 5 V) applied at 2 μs intervals. (e) Pickup coil potentials in MS1 and MS2 (*E*_coil1_ and *E*_coil2_) measured upon application of *P_e_* without a sample.

Figure 1 shows a set of diagrams for the magneto sensor system used in this study. The gradio-magneto sensor device (a) is made of ordinary electro-magnetic materials, and is operated at room temperature. Thus, biomagnetic fields that closely approach those of samples can be measured (b). Gradio-magneto sensors require a pair of detectors for both the biological sample and environmental magnetic fields (*M*_b+e_), and for the environmental magnetic field alone. MS1 and MS2 are detectors for the former and the latter, respectively (a,c). Subtraction of each output removes environmental magnetic noise, including geomagnetism. We have improved the gradio-magneto sensor device by using a continuous single CoFeSiB amorphous (Am) wire with a pair of transducer coils mounted on both ends. Thus, unlike the gradio-magneto sensor device previously made by a pair of magneto-impedance elements^6^,^7^, this device is physically inseparable. Application of an excitation-pulse (*P*_e_) (d) induces induction potentials in the transducer coils of MS1 and MS2, which are similar in amplitude and decay time course (e) in the absence of the sample magnetic field. This is ascribed to the symmetrical magnetic field towards both ends of the wire. Also, it is noted that although this sensor uses an analogous device of a magneto-impedance element (i.e. a transducer coil with an amorphous metal wire), it does not measure the impedance of the amorphous wire during application of an AC current, but measures the amplitude of the induction potential in transducer pickup coils upon application of an excitation pulse (For more details see Methods, and Fig. S1).

**Figure 2:**
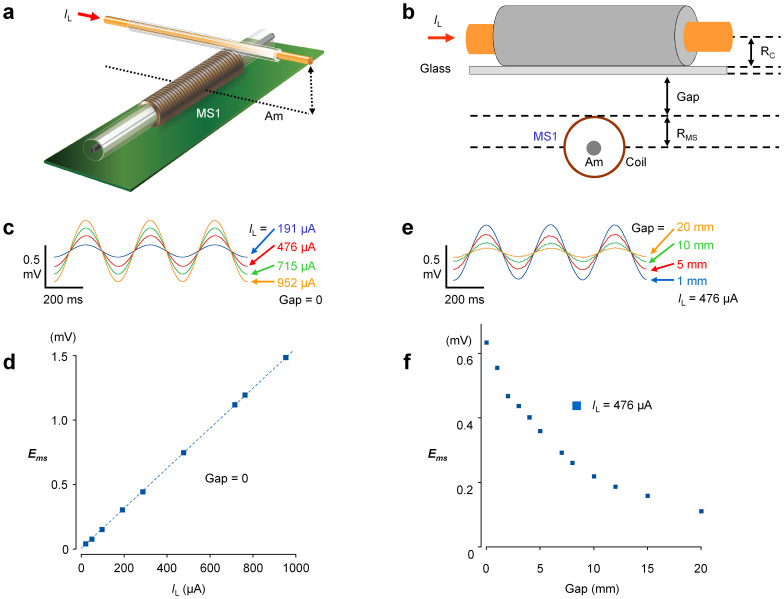
Detection efficacy of the improved gradio-magneto sensor system with a continuous Am wire. (a,b) A linear cable (30 cm in length, 0.5 mm in radius: R_c_) in which a current generator provides oscillating sine waves of 3 Hz. Various amplitudes of the linear cable current (*I*_L_) were applied, and the linear cable was raised with various gaps. (c,d) Changes in the output voltage of the magneto sensor amplifier (*E*_ms_) by applying various *I*_L_ with no gaps. (e,f) Changes in *E*_ms_ by shifting the *I*_L_ gap distance by 476 μA.

Figure 2 shows the determination of specifications for the gradio-type magneto sensor with a continuous single amorphous wire. An insulated electric cable (1 mm in diameter; 30 cm in length) is placed at the center of MS1 (a, b), and the amplitude of the current applied to the cable and the gap between MS1 and the cable is changed (c–e). The output potential of the magneto sensor increased in proportion to the electric current amplitude (R = 1.00), indicating a linear voltage conversion of the objective magnetic field. Also, as the gap between MS1 and the cable increased (gap distance), the output decreased inversely (f). From the relationship between the output potential and gap distance, the sensitivity of the magnetic field was estimated to be ~25 μV/nT.

### Biomagnetic field measurement

**Figure 3:**
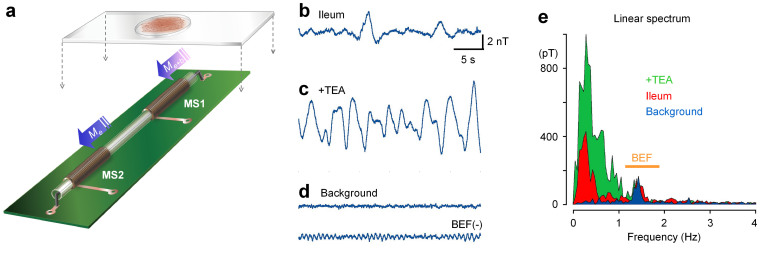
Quasi-real-time measurements of biomagnetic fields from an ileal musculature. (a) The sample was fixed in a recording chamber on MS1. MS1 detects biomagnetic fields along with environmental magnetic fields, while MS2 detects only the latter. Environmental magnetic fields were canceled by subtracting the MS2 signal from MS1 signal. (b) An example of a real-time measurement of biomagnetic activity in an ileal musculature sample in normal solution. The sample was mounted with the longitudinal muscle layer down, and perpendicular to the MS1. (c) Spontanoous biomagnetic activity was applified by application of TEA (0.5 mM). A band elimination filter (BEF) was applied in off-line analysis in (b) and (c). (d) Background noise traces with and without BEF. (E) Linear spectra of b, c and d. The yellow line represents the frequency range of BEF applied in (b–d).

Biomagnetic fields were measured in musculatures isolated from guinea-pigs without using any magnetic shield. The sample was fixed in a recording chamber on MS1 (Fig. 3a). Environmental magnetic noise was removed by subtracting the MS2 signal from that of MS1 in this magneto sensor system. Also, during biomagnetic field measurements, the recording chamber and the near-by magneto sensors were kept at 34–36°C, using a plastic panel heater. Panel b (Fig. 3b) shows an example of recording spontaneous magnetic activity in an ileal musculature mounted with the longitudinal muscle layer down and perpendicular to MS1 in the recording chamber. Application of a K^+^ channel blocker (0.5 mM tetraethyl ammonium: TEA) significantly enhanced the magnetic activity (c), in accordance with its excitatory effects on electric activity^8^. The active magnetic fields observed are considered to reflect the ability to propagate electric activity in electric syncytia of the musculature. The gradio-magneto sensor measurements usually contained a low frequency noise of ~1.4 Hz (d, upper trace), but a band elimination filter effectively reduced the background noise (d, lower trace). Linear spectra (e) and histograms (Fig. S2) extracted the amplification features of biomagnetic fields in the presence of TEA, compared with the background noise. Since dihydropyridine Ca^2+^ channel antagonists nearly completely abolished the response to TEA (Fig. S3), it was suggested that the enhancement of magnetic activity employed electric currents in smooth muscle cells.

### Distance and direction of the sample

**Figure 4:**
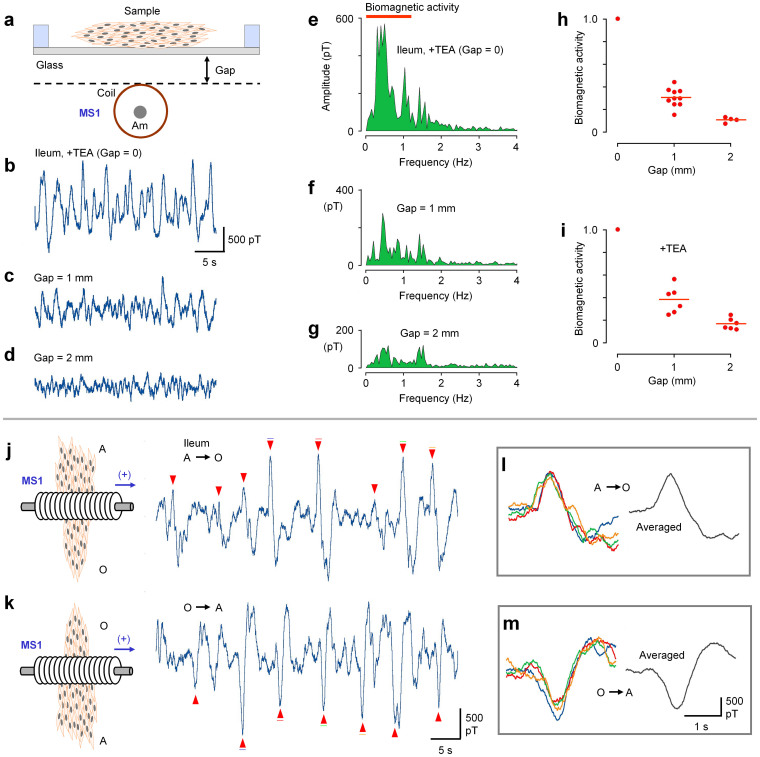
Biomagnetic fields characterized by gap distance (a–i) and direction of musculature (j–m). (a–d) Gap distance between the cover glass and MS1 magneto sensor was changed from 0 to 1 and 2 mm, in the presence of a K^+^ channel blocker (0.5 mM TEA). (e–g) Linear spectra for the magnetic field recordings B to D. Note that increases in gap distance largely reduced the signals < ~1.2 Hz, while the magneto sensor noise remains at around 1.5 Hz. (h,i) Sum of linear spectrum amplitude in the frequency range indicated in (e) (thick red line) is plotted as biomagnetic activity against gap distance, relative to that without a gap, in the absence and presence of TEA. Thin red lines represent the average of experiments. (j,k) Biomagnetic field measurements from the same ileal musculature that crossed the MS1 magneto sensor in anal-to-oral (A → O) and oral-to-anal (O → A) ends, respectively. Gap = 0. The schema indicates the bottom view of the sample and magneto sensor. (l,m) Four expanded biomagnetic waves in (j) and (k) are superimposed (left) and averaged (right) in (l) and (m), respectively. Biomagnetic waves used are indicated by color bars in (j) and (k).

The magnitude of a magnetic field is reduced depending on the distance between the sensor and electric current source. We thus examined the effects of gap distance between the sample musculature and MS1, as shown in Fig. 4a. As the gap distance increased to 1 and 2 mm, the biomagnetic waves of ileal musculatures decreased rapidly, as seen in magnetic field traces (b–d) and in linear spectra (e–g) measured in the presence of TEA. Changes in the biomagnetic field were quantified by integrating a linear spectrum in the range of <1.2 Hz, avoiding low frequency noise. The magnitude of the biomagnetic field decreased to 30.3 ± 8.2% (n = 10) and to 10.5 ± 2.4% (n = 4) when the gap distance was 1 and 2 mm, respectively, in normal solution (h), and it decreased to 37.9 ± 12.0% (n = 6) and 16.6 ± 5.0% (n = 6), when the gap distance was 1 and 2 mm, respectively, in the presence of TEA (i).

The direction of magnetic field changes depended on the propagating direction of the electric current. Thus we assessed the effect of reversing the direction of the musculature sample on MS1 (the rotation of the sample and MS1) on the signal output (Fig. 4j–m). In j, an ileal musculature crossed MS1 from the anal-to-oral ends, observed from the bottom side of the sensor. The magneto sensor recorded many magnetic waves consisting of an upstroke component (red arrow head) followed by a downstroke component. Subsequently, the direction of the ileal musculature was reversed, crossing MS1 from the oral-to-anal ends, as shown in k. This procedure reversed the direction of magnetic waves. Four large magnetic waves in j and k are shown expanded in l and m, respectively, clearly indicating the reverse of biomagnetic fields. Since positive (upward) signals in the sensor output represent the rightward direction of the magnetic field (MS2 is placed to the left of MS1; positive signals represent the direction of MS2 to MS1), the magnetic waves in Fig. 4j–m correspond to a propagating electric current in the oral-to-anal direction in this sample.

The effect of reversing the musculature direction was also examined in stomach samples, because the propagation direction of electric activity appears to persist for a long time^8^: Each reversed direction of the musculature sample changed the direction of the magnetic field (Fig. S4). Essentially similar results were obtained in other ileal (n = 5) and gastric musculature (n = 4) samples. These results reinforce that gradio-magneto sensors can detect magnetic fields of a propagating electric current in the syncytia of musculatures, but are not significantly influenced by musculature vibrations^9^, which may affect the susceptibility of amorphous metal wires through magnetostrictive effects.

### Simulations of biomagnetic fields

**Figure 5:**
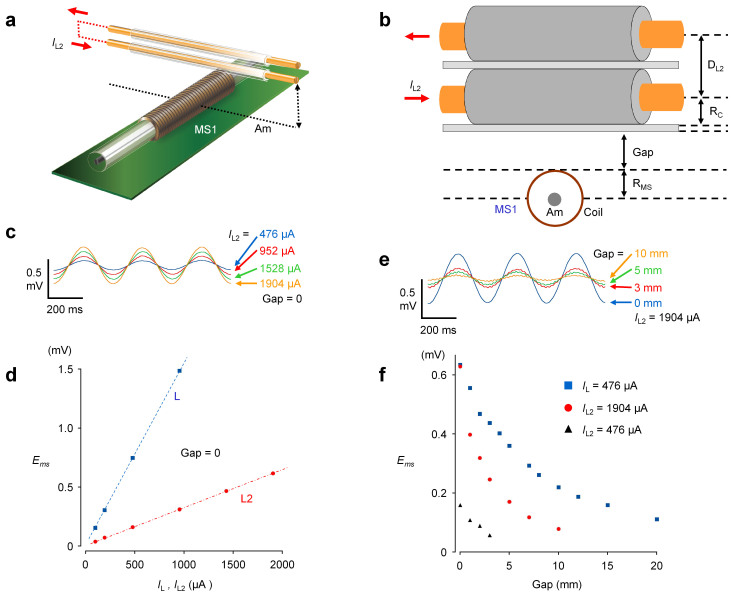
Practical model of a biomagnetic field. (a,b) A pair of linear insulated electric cables (30 cm in length, 0.5 mm in radius: R_c_) are piled on top of an MS1 magneto sensor and separated by a cover glass plate (100 μm thick). The distance between the two cables (*D*_L2_) is ~1.1 mm. Various amplitudes of current (*I*_L2_) were applied to the paired cables, which were raised with various gaps. (c,d) Changes in the output voltage of the magneto sensor amplifier (*E*_ms_) by applying various *I*_L2_ with no gaps. (e,f) Changes in *E*_ms_ by shifting the *I*_L2_ gap distance by 1904 μA. The results of single cable (Fig. 2 d and f: *I*_L_ = 476 μA) are reproduced to show the difference. Also, in (f), changes in *E*_ms_ with an *I*_L2_ of 476 μA are shown.

The biomagnetic field decreased rather rapidly as gap distance increased (Fig. 4h,i), compared with the magnetic field measurements with a single electric cable. To account for this, we assumed that the electric current propagated in the musculature sample was accompanied by a return current, with an effective distance between these currents of several hundred μm. Figure 5 shows magnetic field measurements from a practical model in which two electric cables (1 mm diameter; 30 cm in length) are piled one upon the other separated by a thin glass (100 μm) (a,b). The output of the magneto sensor also increased in proportion to the electric current amplitude applied (R = 1.00), but the gradient was significantly smaller than that examined in a single electric cable (3.2 *vs* 15.5 V/A) (c,d). Also, the gap distance decreased the signal output in two parallel cables more effectively than in a single cable. The amplitude was reduced to ~27% at 5 mm.

**Figure 6:**
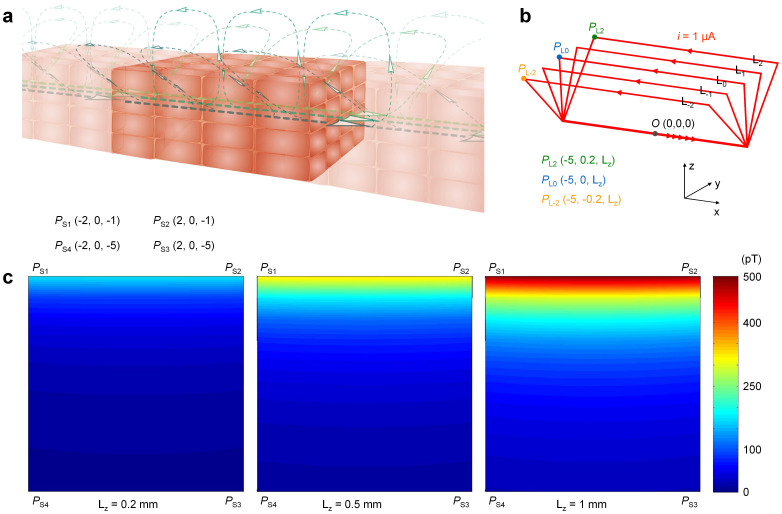
Computer simulation of a biomagnetic field. (a) An illustration of cellular organizations with intercellular propagating current and extracellular return current. The return current may consist of intercellular currents towards surface cells as well as extracellular surface currents. (b) Simplified circuits for simulation of a biomagnetic field. Five electric circuits (L_−2_ to L_2_) are combined and a 1 μA current is conducted in each circuit. In brackets, x, y and z coordinates of points in the circuit are indicated in mm. The distance of current propagation corresponds to ~60 cells in a longitudinal direction, assuming a cell length of 150 μm. (c) Biomagnetic field maps of L_z_ = 0.2, 0.5 and 1 mm. *P*_S1_ – *P*_S4_ are the four corners of the map. Note the amplification of the magnetic field by increasing the L_z_ distance of the circuit.

Due to the limitations of a lab-made practical model in which the length of electric current segments vertical to the sensor are significantly longer than those of in parallel (see more detailed explanation in the Appendix in SI), we next assessed the distribution of the biomagnetic field using computer models (Fig. 6). We assumed the propagating intercellular current and return extracellular current distribution by simplifying with five combined rectangular circuits (a,b). The pseudo color maps display that the magnetic field decreases rapidly as the distance from the rectangular circuit increases; for example, it decreased to ~30% between 1 and 2 mm from the circuit. The magnetic field was amplified by increasing the loop distance (c). As shown in the practical and computer models (Figs. 5 and 6; Figs S5–S7), the rapid reduction of biomagnetic fields with a small distance can be attributed to the features of biological electric circuits constructed by intercellular and transmembrane ionic conductions. Namely, a major propagating intercellular current and return extracellular currents are separated by a small distance, at least less than the thickness of samples. In other words, the size of an effective circuit for generating a biomagnetic field is rather small, presumably in the range of several hundreds of μm to several tens of mm (within the size of electric syncytium at most).

## Discussion

The musculature of the gastrointestinal tract is known to act as a group of electric syncytia to achieve its function. When a part of the syncytium receives chemical or electric stimuli, electric signals propagate through intercellular electric connections, i.e. gap channels, organizing the syncytium and allowing it to respond as a whole. Electric connectivity of gut musculatures has been shown by conventional microelectrodes^10^ and a microelectrode array (MEA)^8^. However, the experiments with microelectrodes were carried out using an electric current injector, while the MEA measurements merely showed spatial synchronizations of electric activity.

In the present study, we detected biomagnetic fields in gut musculatures, using an amorphous metal-based magneto sensor in a quasi-real-time manner (Fig. 3). The measurements demonstrated a vector feature of biomagnetic fields reflecting the propagation direction of spontaneous electric activity (Fig. 4 and Fig. S4). In addition, the amplitude of magnetic waves represents the conductivity of electric activity. These parameters provide us with new functional information of cellular organizations. Furthermore, we observed a rapid reduction of the magnitude of magnetic activity by increasing the distance between the sensor and sample (Fig. 4): ~30% decreases by the initial and subsequent 1 mm separations. Along with the practical and computer simulations of biomagnetic fields (Figs. 5 and 6), this observation can be attributed to a rather small size (<1 mm thick) of effective electric circuits in the cellular organizations examined in the present study. This feature of a bioelectric circuit can also account for the limitations of measurements in small biological samples using a SQUID whose detector coils are placed in a liquid container^11^. In line with this deduction, previous volume conductor models for an isolated whole heart, have displayed a slower reduction of magnetic fields (from 1 nT to 100 pT with a separation of ~10 mm)^12^.

Under control conditions, the amplitude of magnetic waves measured in ileal musculatures was within the range of several nT, when the MS1 sensor was placed below the recording chamber (Fig. 3). Since MS1 detectors and samples are separated by a cover glass of ~100 μm, and the amorphous wire of MS1 (= MS2) is surrounded by a transducer coil with a 500 μm radius, the total distance between the magneto sensor (the center of the amorphous wire) and the propagating major intercellular current is assumed to be ~1 mm. On the other hand, the computer models with a distance of 0.2–0.5 mm between intercellular currents and return currents (Fig. 6 c, left and center maps) better reflect ileal musculatures. In the maps of these models, the total circuit current was 5 μA, and the magnetic field at 1 mm below the intercellular current is estimated to be ~200–300 pT. Also, previous sucrose-gap voltage-clamp experiments and theoretical analysis indicate that a small length (0.5 mm) of gut musculature requires a transmembrane electric current of more than several to several tens of μA at depolarizations corresponding to spike activities^13^,^14^. Therefore, the magnitude of biomagnetic fields observed in this study (up to several nT) may correspond to electric activities of only a part of musculature samples (25 mm long × 5 mm wide). In future studies, we may need to consider this issue (the ratio of active region) by using a magneto sensor with a higher spatial resolution, such as a multi-channel amorphous metal-based magneto sensor, and by using more accurate models of an effective circuit (Fig. S7).

Special pacemaker cells are known to exist in the ileum, and play an essential role in generating spontaneous electric activity. From histological features, the pacemaker cells are referred to as interstitial cells of Cajal (ICC)^15^,^16^. However, in light of the distinct expression of ion channels, the TEA facilitation of biomagnetic waves is ascribed to an electric current in the smooth muscle. 1) In smooth muscle cells, TEA blocks a major K^+^ current, and also amplifies intracellular Ca^2+^ transients, both being known to enhance excitability^17^,^18^,^19^. On the other hand, a TEA-insensitive K^+^ current plays a major role in regulating pacemaker activity in ICC^20^. 2) Upon depolarization, L-type Ca^2+^ channels (Ca_v_1.2) are responsible for a voltage-gated inward current in smooth muscle^21^,^22^, while T-type Ca^2+^ channels (Ca_v_3.2) as well as TTX-insensitive voltage-gated Na^+^ channels are suggested to play a major role in ICC pacemaking^23^,^24^. The fact that application of nifedipine, an L-type Ca^2+^ channel-specific blocker, abolishes biomagnetic waves in the presence of TEA (Fig S3), reinforces a predominant contribution of smooth muscle electric current to intercellular electric current under normal conditions. It is considered that although ICC pacemaker current underlies smooth muscle spontaneous activity^25^,^26^, a major component of electric current propagating through the muscle layer is regenerated in smooth muscle cells.

To date, SQUID has been employed as a major tool to measure biomagnetic fields, such as magnetoencephalography and magnetocardiography^4^,^27^,^28^,^29^. Recently, atomic magnetometers utilizing vapors of polarized alkaline metals (Rb, Cs and K) have become popular as an alternative tool^5^,^30^,^31^. These two magnetometers are currently available for biomagnetic field measurements. The former is a vector magnetometer, while the latter is a scalar magnetometer. Therefore, only the former SQUID magnetometer is available for measuring biomagnetic vector fields, similar to the present measurements. Since the former and latter are normally operated at extremely low and high temperatures, respectively, compared to body temperature, sensor devices are mounted in containers separated from biological systems. SQUID microscopy with small pickup coils (500 μm in diameter) has been applied to an isolated whole heart to measure electric stimulation-evoked magnetic fields with a sub-pT range sensitivity^32^,^33^. More recently, atomic magnetometers operated at a room temperature have been reported, but sensitivity is similarly reduced^34^. Also, both magnetometers require a magnetic shield to reduce the environmental magnetic field within a maximal detection limit. Therefore, the total system becomes large in these magnetometers.

In the light of the present experiments, we propose a new option to measure biomagnetic fields, depending on the aim and objective of study. The gradiomagneto sensor device used herein is made of an amorphous metal wire and ordinary electro-magnetic materials, and is operated at ambient temperature. Thus, our sensor can be placed very close to the sample, and can also detect the magnetic fields of a limited region that corresponds to the volume of the amorphous metal wire. On the other hand, magnetometers with very high sensitivity, such as SQUID and atomic magnetometers, have the same average volume of detector coils and vapor containers, respectively. Also, amorphous metal-based sensors do not require any magnetic shield against geomagnetic fields, because voltage conversion of the magnetic field is linear over a range of ±50 μT^9^. For these reasons, our magneto sensor is applied to measure small biological samples, and can be compacted for portable use.

Details of single cell and single ionic channel properties are now being elucidated due to the development of micromanipulation and fine voltage-clamp technologies^35^. In contrast, electric properties of cellular organizations remain unknown. Since innumerous electric syncytia of excitable cells with intercellular electric coupling, exist over the entire human body, such information enables the evaluation of integrated functions of biological systems. The magneto sensor used in this study could be a good tool for such a purpose. Also, in regenerative medicine, magneto sensors with high sensitivity may be useful for aseptic and non-invasive evaluations of spatial activity in cellular tissue samples derived from stem cells^36^,^37^. Especially, for small samples of biological systems, magneto sensors that can closely approach the sample are advantageous to detect magnetic fields, because biomagnetic fields induced in small circuits rapidly decline even over a small distance (Fig. 6c).

The sensitivity and stability of amorphous material-based magnetic sensors are expected to improve further with additional development^38^. The intrinsic noise of the sensor is estimated to be less than 10 fT/√ (Hz) in the same sized wire used in this study, assuming the electron spin density of a typical Co-rich amorphous metal without a significant magnetic domain movement^39^,^40^,^41^. Therefore, in order to improve this magneto sensor, it is crucial to use uniformly manufactured paired transducer coils designed for amorphous metal materials, and to find good amorphous metal materials and electric devices that more efficiently convert excitation-pulse-induced induction potentials into the signal output of a magnetic field^42^. Also, amorphous metal-based magneto sensors could be developed as micro-electro mechanical system (MEMS)-like integrated circuits, thus allowing the total apparatus to be compacted for use in small labs, hospitals and even for home use, without using any magnetic shield. In initial studies of biological electric activity, such as electrocardiograms, measurements were carried out using a galvanometer, which converts changes in a magnetic field to induction potentials, a remind that measurements of biological magnetic and electric fields are closely related. Unfortunately, at present, biomagnetic field measurements are performed at only a few sites, while portable bioelectric detectors are available in electrocardiography and electroencephalography. We anticipate that amorphous metal-based magneto sensor technology would make biomagentic fields a more realistic aspect in our lives, and that this technology could be employed to make new detectors for biological and medical samples, depending on the aim and objective of the study.

## Methods

### Magneto sensor

The set of panels in Fig. 1 shows the amorphous metal-based gradio-magneto sensor system used in the present study. To improve the gradio-sensing of a magnetic field, a pair of transducer solenoid coils (0.5 mm in radius; 10 mm in length; 300 turns) was mounted at both ends of a single amorphous (Am) wire (30 μm in diameter, ~50 mm in length) of no magnetostriction. The composition of the wire used was (in atomic percentages): Co-F alloy (94 *vs* 6) 72.5; Si 12.5; B 15. Presumably, due to the symmetrical magnetic fields distributed along the amorphous wire, the induction potentials in the paired transducer coils were much more identical in terms of the amplitude and decay time course, compared with those induced in a pair of separated magneto-impedance (MI) elements previously used^6^,^7^.

The driving and detecting devices were as follows: A clock CMOS IC triggers a pulse gate (PG) CMOS IC that supplies electric excitation pulses (*P*_e_) of 5 V (100 ns) to the magnetic amorphous wire at 2 μsec intervals. The intermediate portion of the wire was electrically shunted with an electric cable in order to reduce the resistance of the excitation circuit to ~50 Ω. The same clock IC simultaneously triggers a pair of sample-and-hold circuits (SH1, SH2) to detect the induction potential of the coils in MS1 and MS2 magneto detectors. The detection devices for the paired coils were essentially similar to those used previously^6^,^7^ (See Fig S1 for magneto detection mechanisms).

High-speed operation amplifiers with a frequency range of several MHz were used to follow and differentiate the SH1 and SH2 voltages (subtracting the induction potential of SH2 from that of SH1) to cancel environmental magnetic noise. Also, the differentiated signal was amplified ~1000 times. After the operation amplifiers, high and low cut electric filters (H/L filter: 20 and 0.3 Hz, respectively) were applied, and the resulting voltage output was sampled to computer memory through a data logger with a frequency of 1 kHz. Positive (upward) signals in figures represent the direction of the magnetic field from MS2 to MS1.

Specifications of the gradio-type magneto sensor were assessed using a model system. A linear electric cable 1 mm in diameter and 30 cm in length was crossed on MS1 at the center, and oscillating electric currents (sine waves) were applied at 3 Hz (Fig. 2). The magnetic field (output voltage) detected progressively decreased as the distance between the electric cable and MS1 increased. The conversion efficacy of magnetic field into output voltage was estimated to be ~25 μV/nT (~0.031 V/A/m, i.e. B = μ_0_H, and μ_0_ = 1.256 × 10^−6^ T/A/m). The noise level was ~30 pT/Hz^½^. Also, the performance of the subtraction (gradio-sensing) between MS1 and MS2 was assessed by changing the amplitude and frequency of the external magnetic field, when the whole sensor system was placed in a Helmholz coil of 30 cm diameter. The output potentials of SH1 and SH2 differ at most by ~3% at 1–20 Hz.

### Animals and preparations

Animals were treated ethically, in accordance with the guidelines for proper conduct of animal experiments by the Science Council of Japan. All procedures were approved by the Animal Care and Use Committee of Nagoya University Graduate School of Medicine (Permission #23357). Guinea pigs of ~3 weeks after birth were killed by cervical dislocation and exsanguination after deeply anaesthetising with diethyl ether. Smooth muscle tissues were isolated with a pair of fine scissors. For magnetic measurements connective tissue and the mucous membrane were carefully removed using forceps and fine scissors under a binocular microscope (SZ61, Olympus, Tokyo, Japan).

### Measurements of biological magnetic field

Musculatures isolated from the ileum (~5 mm wide × 25–35 mm long × 0.5–1 mm thick) were mounted in a recording chamber with the bottom of a cover glass of 100 μm thick, using a tissue anchor rig made by thin strings (SDH series, Harvard Apparatus Japan, Tokyo, Japan). The chamber contained a ‘normal’ extracellular solution kept at 34–36°C using a plastic panel heater in which warm water was sufficiently circulated before measurements (Fig. 3a).

Changes in magnetic fields were measured along the amorphous wire by discriminating the direction. One of the paired magneto detectors (MS1 in Fig. 1 a) was placed below the recording chamber, while the other (MS2) magneto detector at the other end of the amorphous wire was used to sense the environmental magnetic field. The differentiated potential of MS2 from MS1 after the sample-and-hold circuits was recorded as biomagnetic fields arising from ileal musculature samples.

In some experiments, pharmacological interventions were carried out in order to enhance or suppress biological magnetic (and electric) activity (Figs. 3 and 4; Fig. S3).

### Solutions and drugs

The composition of the ‘normal’ extracellular solution (modified Krebs solution) used, was (in mM): NaCl 125; KCl 5.9; MgCl_2 _1.2; CaCl_2_ 2.4; glucose 11; Tris-HEPES 11.8 (pH 7.4). TEA was purchased from Sigma-Aldrich (St Louis, MO, USA).

### Simulation

A practical model of a biological magnetic field was made with a pair of electric cables (1 mm in diameter; 30 cm in length) with a center distance of ~1100 μm (Fig. 5). Magnetic fields were measured using the gradio-magneto sensor by changing the amplitude of the oscillating electric current and the distance between the cable and MS1.

In computer simulation, the magnetic field in a certain point was estimated using the ‘P and Qm’ software package, by integrating a current-element-induced magnetic field along the electric circuit according to Biot-Savart law (Shift Lock Corp., Kishiwada, Japan). Pseudo-color magnetic field maps were drawn using MATLAB software (MathWorks, Arkansas Garden City, USA).

### Data analysis

Digital band-pass filter, and linear spectrum analysis were performed using commercial add-in software (Kyowa Electronic Instruments, Tokyo, Japan). In histogram analysis, recording data of magnetic fields thinned out by 1/10, and 5000 data points (corresponding to 50 sec) were used. Histograms were constructed with a bin width of 5 pT, and plotted with cumulative curves (Fig. S2).

Numerical data are expressed as means ± S.D. Significant differences were evaluated by paired *t*-tests (*P* < 0.05).

## Supplementary Material

Supplementary Information Supplementary Information

## References

[b1] Szurszewski J. H. Electrical basis for gastrointestinal motility in *Physiology of Gastrointestinal Tract* *2nd edn*. (ed. Johnson, L. R.) 383–422 (Raven, 1987).

[b2] Tomita T. Electrical activity (spikes and slow waves) in gastrointestinal smooth muscle in *Smooth Muscle: An Assessment of Current Knowledge*. (eds. Bülbring, E., Brading, A. F., Jones, A. W. & Tomita, T.) 127–156 (Edward Arnold, 1981).

[b3] Wikswo J. P. & Freeman J. A. Magnetic field of a nerve impulse: First measurements. Science 208, 53–55 (1980). 7361105 10.1126/science.7361105

[b4] Williamson S. J., Lü Z. L., Karron D. & Kaufman L. Advantages and limitations of magnetic source imaging. Brain Topogr. 4, 169–180 (1991). 1793690 10.1007/BF01132773

[b5] Budker D. & Romalis M. Optical magnetometery. Nat. Phys. 3, 227–234 (2007).

[b6] Nakayama S., Atsuta S., Shinmi T. & Uchiyama T. Pulse-driven magnetoimpedance sensor detection of biomagnetic fields in musculatures with spontaneous electric activity. Biosens. Bioelectron. 27, 34–39 (2011). 21741817 10.1016/j.bios.2011.05.041

[b7] Uchiyama T., Mohri K. & Nakayama S. Measurement of spontaneous oscillatory magnetic field of guinea-pig stomach muscle preparation using pico-Tesla resolution amorphous wire magneto-impedance sensor. IEEE Trans. Magn. 47, 3070–3073 (2011).

[b8] Nakayama S. *et al.* Pacemaker phase shift in the absence of neural activity in guinea-pig stomach: a microelectrode array study. J. Physiol. 576, 727–738 (2006). 16990400 10.1113/jphysiol.2006.118893 PMC1890421

[b9] Mohri K. *et al.* Amorphous wire and CMOS-based sensitive micromagnetic sensors utilizing magneto-impedance (MI) and stress-impedance (SI) effects. IEEE Trans. Magn. 38, 3063–3068 (2002).

[b10] Abe Y. & Tomita T. Cable properties of smooth muscle. J. Physiol. 196, 87–100 (1968). 5653888 10.1113/jphysiol.1968.sp008496 PMC1351736

[b11] Tanaka S. *et al.* Measurement of the signal from a cultured cell using a high-Tc SQUID. Supercond. Sci. Technol. 16, 1536–1539 (2003).

[b12] Murdick R. A. & Roth B. J. A comparative model of two mechanisms from which a magnetic field arises in the heart. J. Appl. Phys. 95, 5116–5122 (2004).

[b13] Bolton T. B. Effects of stimulating the acetylcholine receptor on the current-voltage relationships of the smooth muscle membrane studied by voltage clamp of potential recorded by micro-electrode. J. Physiol. 250, 175–202 (1975). 1177118 10.1113/jphysiol.1975.sp011048 PMC1348344

[b14] Tomita T. Membrane capacity and resistance of mammalian smooth muscle. J. Theoret. Biol. 12, 216–227 (1966). 5972192 10.1016/0022-5193(66)90114-7

[b15] Sanders K. M. *et al.* Development and plasticity of interstitial cells of Cajal. Neurogastroenterol. Motil. 11, 311–338 (1999). 10520164 10.1046/j.1365-2982.1999.00164.x

[b16] Furness J. in The enteric nervous system 1st edn. 1–288 (Wiley-Blackwell, 2006).

[b17] Muraki K. *et al.* Effects of tetraethylammonium and 4-aminopyridine on outward currents and excitability in canine tracheal smooth muscle cells. Br. J. Pharmacol. 100, 507–515 (1990). 1697197 10.1111/j.1476-5381.1990.tb15838.x PMC1917802

[b18] Farrugia G., Rae J. L. & Szurszewski J. H. Characterization of an outward potassium current in canine jejunal circular smooth muscle and its activation by fenamates. J. Physiol. 468, 297–310 (1993). 8254511 10.1113/jphysiol.1993.sp019772 PMC1143827

[b19] Borisova L., Shmygol A., Wray S. & Burdyga T. Evidence that a Ca^2+^ sparks/STOCs coupling mechanism is responsible for the inhibitory effect of caffeine on electro-mechanical coupling in guinea pig ureteric smooth muscle. Cell Calcium 42, 303–311 (2007). 17298845 10.1016/j.ceca.2006.12.005

[b20] Zhu Y. *et al.* ERG K^+^ currents regulate pacemaker activity in ICC. Am. J. Physiol. Gastrointest. Liver Physiol. 285, G1249–1258 (2003). 12958021 10.1152/ajpgi.00149.2003

[b21] Nakayama S. *et al.* Tyrosine kinase inhibitors and ATP modulate the conversion of smooth muscle L-type Ca^2+^ channels toward a second open state. FASEB J. 20, 1492–1494 (2006). 16738256 10.1096/fj.05-5049fje

[b22] Akbarali H. I., Hawkins E. G., Ross G. R. & Kang M. Ion channel remodeling in gastrointestinal inflammation. Neurogastroenterol. Motil. 22, 1045–1055 (2010). 20618833 10.1111/j.1365-2982.2010.01560.x PMC2939949

[b23] Strege P. R. *et al.* Effect of mibefradil on sodium and calcium currents. Am. J. Physiol. Gastrointest. Liver Physiol. 289, G249–253 (2005). 15790762 10.1152/ajpgi.00022.2005

[b24] Gibbons S. J. *et al.* The alpha1H Ca^2+^ channel subunit is expressed in mouse jejunal interstitial cells of Cajal and myocytes. J. Cell. Mol. Med. 13, 4422–4431 (2009). 19413888 10.1111/j.1582-4934.2008.00623.x PMC2855776

[b25] van Helden D. F. & Imtiaz M. S. Ca^2+^ phase waves: a basis for cellular pacemaking and long-range synchronicity in the guinea-pig gastric pylorus. J. Physiol. 548, 271–296 (2003). 12576498 10.1113/jphysiol.2002.033720 PMC2342787

[b26] Sperelakis N. & Daniel E. E. Activation of intestinal smooth muscle cells by interstitial cells of Cajal in simulation studies. Am. J. Physiol. Gastrointest. Liver Physiol. 286, G234–243 (2004). 14715518 10.1152/ajpgi.00301.2003

[b27] Cohen D., Norman J. C., Molokhia F. & Hood W. Jr Magnetocardiography of direct currents: S-T segment and baseline shifts during experimental myocardial infarction. Science 172, 1329–1333 (1971). 5580214 10.1126/science.172.3990.1329

[b28] Koch H. Recent advances in magnetocardiography. J. Electrocardiol. 37 Suppl, 117–122 (2004). 15534820 10.1016/j.jelectrocard.2004.08.035

[b29] Soekadar S. R. *et al.* In vivo assessment of human brain oscillations during application of transcranial electric currents. Nat. Commun. 4, 2032; 10.1038/ncomms3032 (2013). 23787780 PMC4892116

[b30] Kamada K., Ito Y. & Kobayashi T. Human MCG measurements with a high-sensitivity potassium atomic magnetometer. Physiol. Meas. 33, 1063–1071 (2012). 22621881 10.1088/0967-3334/33/6/1063

[b31] Johnson C. N., Schwindt P. D. & Weisend M. Multi-sensor magnetoencephalography with atomic magnetometers. Phys. Med. Biol. 58, 6065–6077 (2013). 23939051 10.1088/0031-9155/58/17/6065 PMC4030549

[b32] Baudenbacher F., Peters N. T., Baudenbacher P. & Wikswo J. P. Jr High resolution imaging of biomagnetic fields generated by action currents in cardiac tissue using a LTS-SQUID microscope. Physica C 368, 24–31 (2002).

[b33] Baudenbacher F., Peters N. T. & Wikswo J. P. Jr High resolution low-temperature superconductivity superconducting quantum interference device microscope for imaging magnetic fields of samples at room temperatures. Rev. Sci. Instrum. 72, 1247–1254 (2002).

[b34] Sander T. H. *et al.* Magnetoencephalography with a chip-scale atomic magnetometer. Biomed. Opt. Express 3, 981–990 (2012). 22567591 10.1364/BOE.3.000981 PMC3342203

[b35] Hamill O. P. *et al.* Improved patch-clamp techniques for high-resolution current recording from cells and cell-free membrane patches. Pflügers Arch. 391, 85–100 (1981). 6270629 10.1007/BF00656997

[b36] Nelson T. J., Martinez-Fernandez A. & Terzic A. Induced pluripotent stem cells: developmental biology to regenerative medicine. Nat. Rev. Cardiol. 7, 700–710 (2010). 20956984 10.1038/nrcardio.2010.159

[b37] Itzhaki I. *et al.* Modelling the long QT syndrome with induced pluripotent stem cells. Nature 471, 225–229 (2011). 21240260 10.1038/nature09747

[b38] Vazquez M. *et al.* On the state-of-the-art in magnetic microwires and expected trends for scientific and technological studies. Phys. Status Solidi A 208, 493–501 (2011).

[b39] Melo L. G. C. *et al.* Optimization of the magnetic noise and sensitivity of giant magnetoimpedance sensors. J. Appl. Phys. 103, 033903; 10.1063/1.2837106 (2008).

[b40] Ding L. *et al.* Equivalent magnetic noise limit of low-cost GMI magnetometer. IEEE Sensors J. 9, 159–168 (2009).

[b41] Gudoshnikov S. *et al.* Highly sensitive magnetometer based on the off-diagonal GMI effect in Co-rich glass-coated microwire. Phys. Status Solidi A 211, 980–985 (2014).

[b42] Uchiyama T. & Nakayama S. Magnetic sensors using amorphous metal materials: Detection of premature ventricular magnetic waves. Physiol. Rep. 1, e00030; 10.1002/phy2.30 (2013). 24303116 PMC3831925

