# Supplementary material for: Real-time Measurement of Biomagnetic Vector Fields in Functional Syncytium Using Amorphous Metal
Source: Sci Rep. 2015 Mar 6;5:8837. doi: 10.1038/srep08837 (PMC4351545; doi:10.1038/srep08837)
Supplement: Supplementary Information [file srep08837-s1.docx]

**SUPPLEMENTAL INFORMATION**

**Real-time Measurement of Biomagnetic Vector Fields in Functional Syncytium Using Amorphous Metal**

**By**

Shinsuke Nakayama* and Tusyoshi Uchiyama†

**From**

*Department of Cell Physiology, Nagoya University Graduate School of Medicine, Nagoya 466-8550, Japan.

†Department of Electronics, Nagoya University of Graduate School of Engineering, Nagoya 464-8603, Japan

***Corresponding author**: Shinsuke Nakayama

Department of Cell Physiology, Nagoya University Graduate School of Medicine,

65 Tsuruma-cho, Showa-ku, Nagoya 466-8550, JAPAN

Phone: +81 52 744 2045 Fax: +81 52 744 2048

E-mail: [h44673a@nucc.cc.nagoya-u.ac.jp](mailto:h44673a@nucc.cc.nagoya-u.ac.jp)

**List of Supplemental Information:**

Figure S1: The principle of magneto sensor.

Figure S2: Amplitude histograms.

Figure S3: Inhibitory effect of nifedipine on biomagnetic waves.

Figure S4: Changes in biomagnetic field direction depending on the direction.

Figure S5: Computer simulation of a biomagnetic vector field.

Figure S6: Computer simulation of a biomagnetic vector field shown in logarithmic scale.

Figure S7: Changes in magnetic field depend on the z-distance in the two sets of model circuits.

Appendix: A simple model circuit for estimating biomagnetic fields.


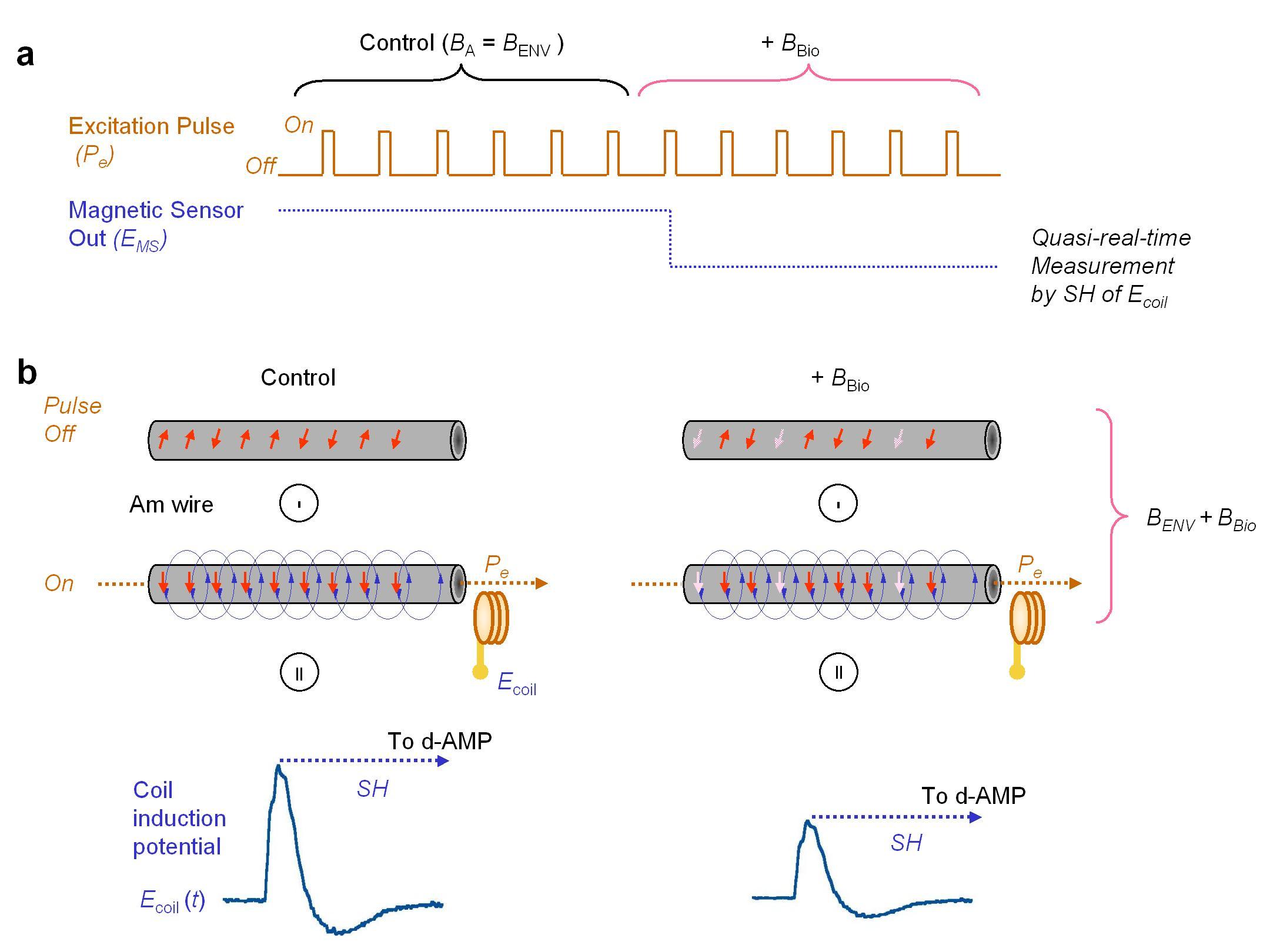


**Figure S1 |** **The principle of a magneto sensor**. Panels show how a magnetic field along the wire axis (*B_A_*) is converted to a coil potential (*E*_coil_) in a pulse-driven magneto sensor constructed with a magnetic amorphous wire (Am) and a transducer coil. (**a**) The periods of control (environmental magnetic fields: *B_ENV_*) and biomagnetic fields (*B_BIO_*) added during excitation pulses were repeated at the same intervals (2 μs). (**b**) Internal magnetization of Am wire and changes in *E*_coil_. Every excitation pulse (*P*_e_) shifts the texture of internal magnetizations to a certain state and the distribution of internal magnetizations returns to an initial state after cessation of *P*_e_ during pulse off time. Thus, the peak amplitude of *E*_coil_(*t*) reflects changes in magnetizations in the presence of a biomagnetic field (pink). A sample/hold (SH) circuit detects the biomagnetic field in a quasi-real-time manner, because of the short interval of *P*_e_ repetition.

**Supplemental Explanation of Magneto Detection Mechanisms in Figure S1.** The exact mechanisms by which pulse-driven amorphous metal-based magneto sensors detect a magnetic field with high sensitivity, are not fully understood, but the principle of the underlying mechanisms currently considered is as follows (Figure S1). 1) Unlike ordinary metals, magnetic amorphous wires of a non-crystalline structure contain internal magnetizations of electron spins whose texture is easily shifted by exposing them to external magnetic fields. 2) In our magneto sensor systems operated by an excitation pulse, electric current pulses (*P*_e_) conduct through the amorphous wire at a constant interval of several μs. Thereby, during excitation (pulse-on period), whole internal magnetizations are shifted to a certain texture following the circumferential magnetic field induced by the surface and/or internal excitation current, and the detector coil surrounding the amorphous wire detects induction potentials which are caused by the shift of magnetizations along the wire axis. 3) The amplitude of induction potential thus reflects the external magnetic field as a difference of the texture of internal magnetizations along the wire axis between the electric pulse-on and off periods. When biomagnetic fields are added to environmental geomagnetism, the induction potential of the detector coil reflects the changes in the magnetization texture for the additional biomagnetic fields. (To simplify the explanation of the detection mechanism, magnetic domain structure is not considered in amorphous metal wires.) 4) In the amorphous wire which we use, the time constant for relaxation of internal magnetizations appears to be short enough (< 100 ns: Fig. 1 **f**) to repeatedly apply excitation pulses at intervals of several μs. Thus, the interval of sampling the magnetic field is sufficiently short compared to electric activities in living systems, and enables biomagnetic fields to be detected in a quasi-real-time manner.

**
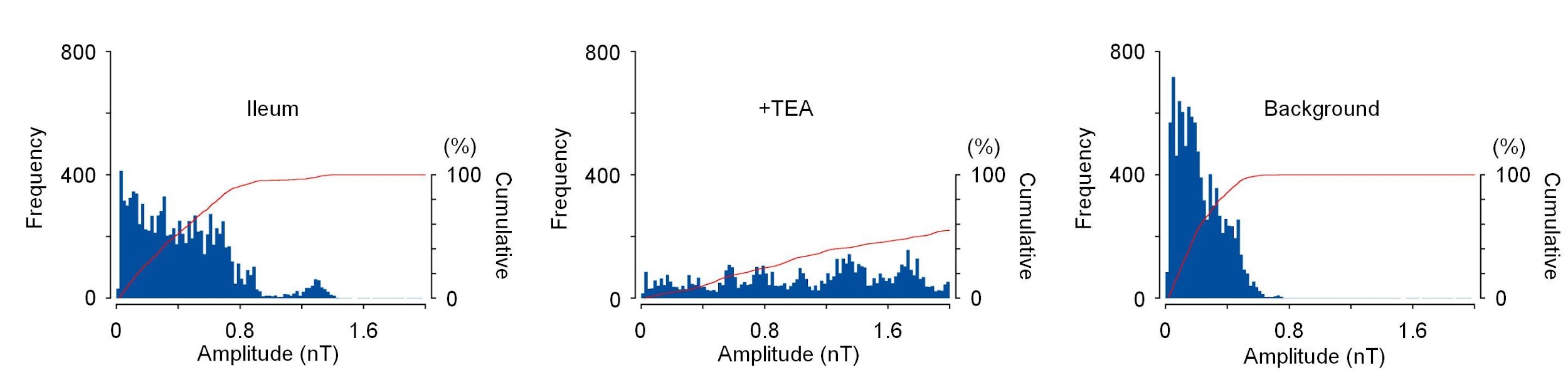
**

**Figure S2 |** **Amplitude histograms.** The recordings of ileal biomagnetic waves in Fig. 3 **b**, **c** and **d** were used, respectively. Off-line band elimination filter (BEF) was not applied. Red curves represent cumulative percentages (%) of the histograms. Bin width = 20 pT. Similar histogram analyses may be useful to quantify the biomagnetic activity in other samples (e.g. use of the amplitude at 50%).
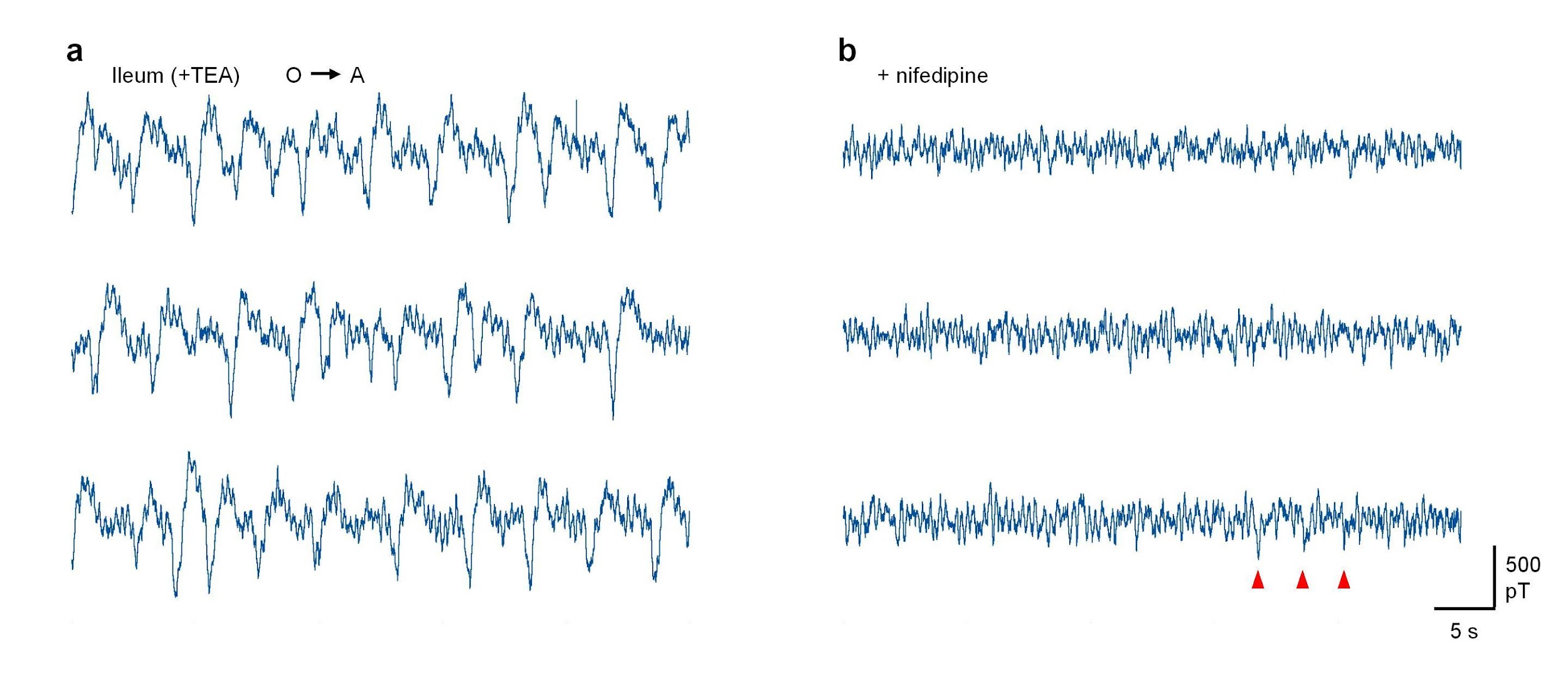


**Figure S3 |** **Inhibitory effect of nifedipine on biomagnetic waves.** After observing biomagnetic waves in an ileal muscular preparation, 1 μM nifedipine, an L-type Ca^2+^ channel blocker, was applied. Panels **a** and **b** show three sequential recordings of biomagnetic waves for ~3 min, before and after application of nifedipine, respectively. The sample was mounted on the chamber in the serosal side down, crossing the magneto sensor from the oral to anal side (O 🡪 A). Note that nifedipine nearly completely suppressed biomagnetic waves, leaving only marginal waves (red arrowheads).


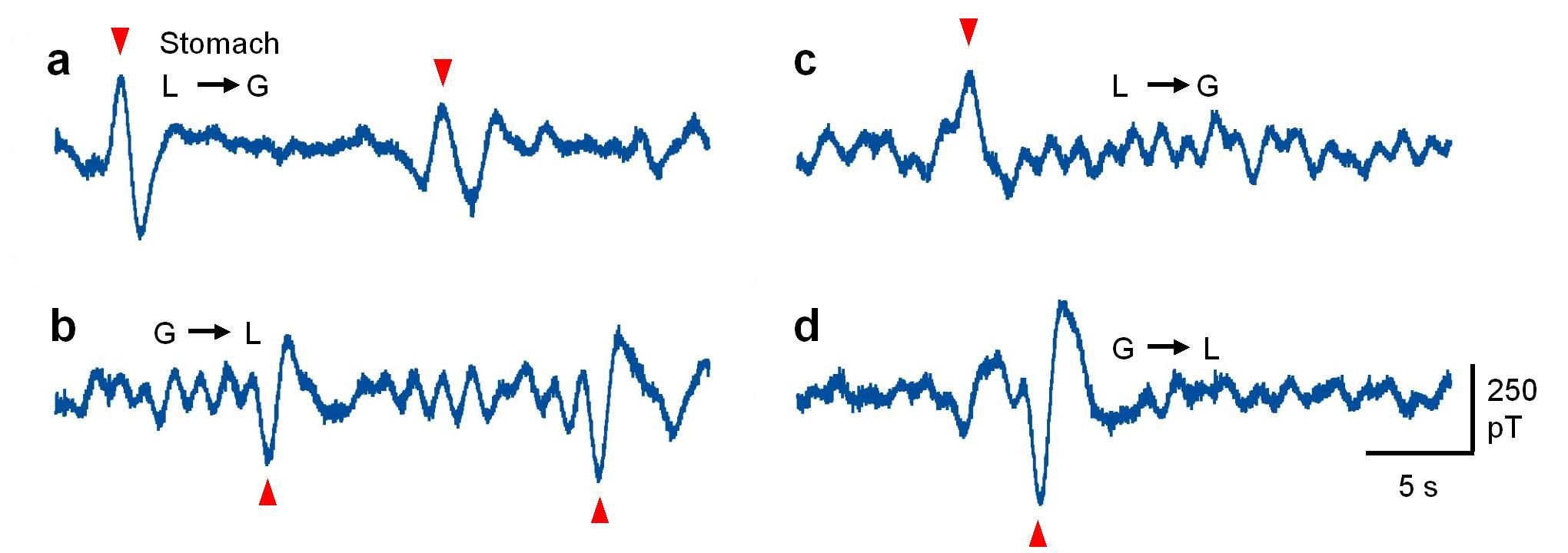


**Figure S4 |** **Changes in biomagnetic field direction depending on the direction of the stomach musculature.** In **a-d**, the direction of the same stomach musculature sample crossing the magneto sensor was alternated in lesser-to-greater (L 🡪 G) and greater-to-lesser (L 🡪 G) curvature ends.

**Figure S5 | Computer simulation of a biomagnetic vector field.** (**a**) The same simplified circuits in Fig. 6 **b** were used for simulation of biomagnetic vector fields. Five electric circuits (*L*_-2_ to *L*_2_) are combined and a 1 μA current is conducted in each circuit. (**b-d**), Biomagnetic vector field maps of L_z_ = 0.2, 0.5 and 1 mm. Top, middle and bottom maps represent x, y and z magnetic vector fields (*M*_x_, *M*_y_, *M*_z_), respectively. Note that *M*_y_ is predominant in this model. To save space, scalar magnetic fields are shown in Fig. 6 **c**.
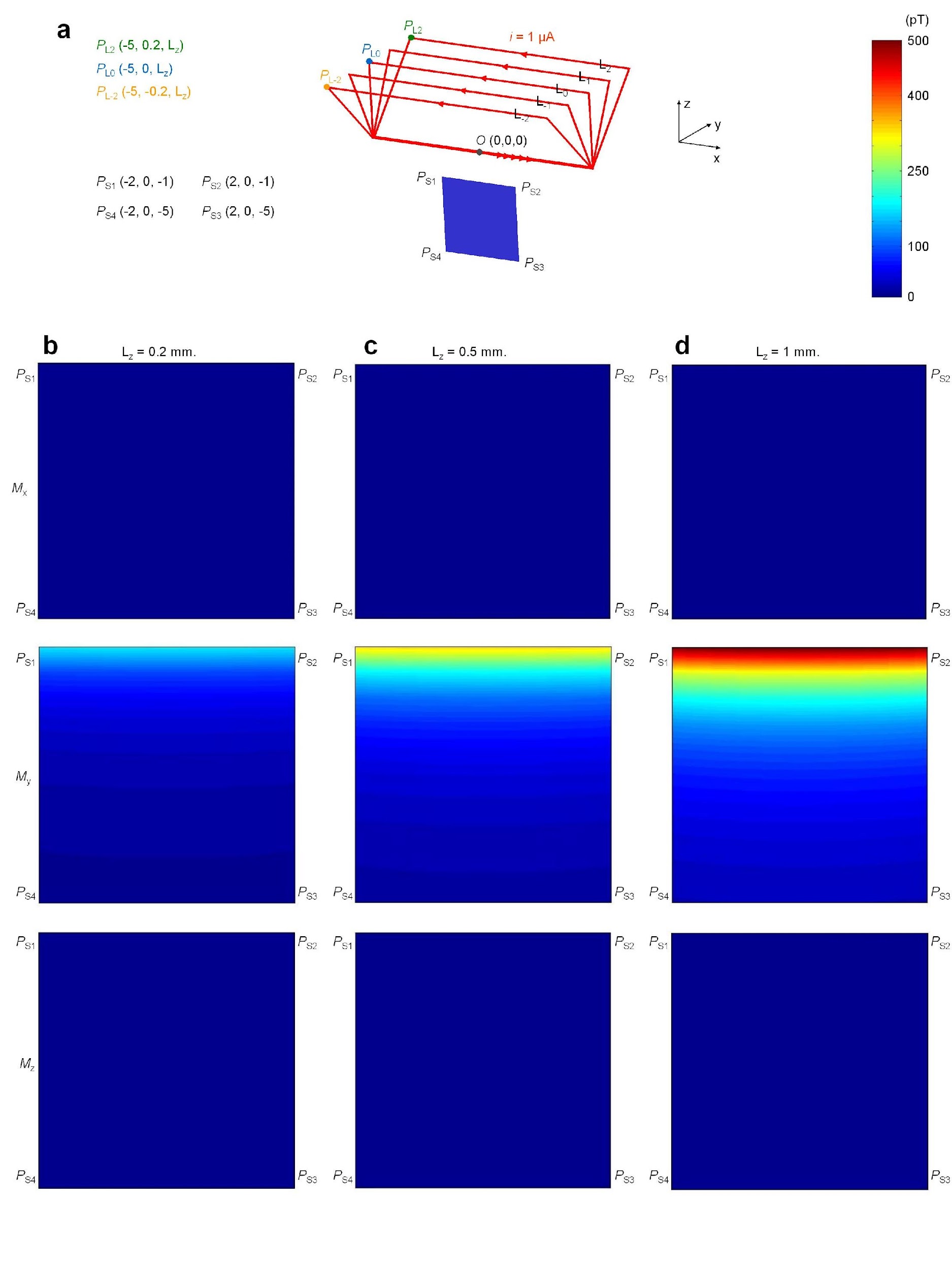


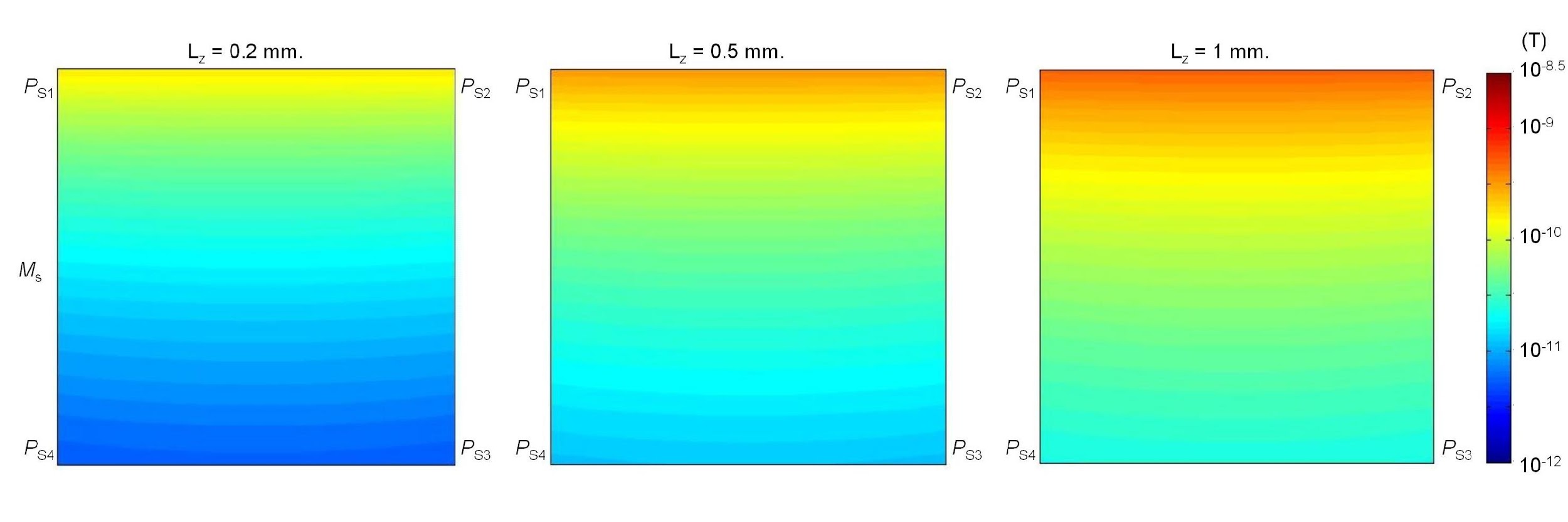


**Figure S6 |** **Computer simulation of a biomagnetic vector field shown in logarithmic scale.** Biomagentic vector field was estimated using the same electric circuits shown in Fig. S5. The y magnetic vector field (*M*_y_) is shown, because of dominant effect.

**Figure S7 |** **Changes in magnetic field depend on the z-distance in the two sets of model circuits.** (**a, b**) The amplitude of a magnetic field is plotted against the z-distance (from 250 μm to 20 mm) toward the bottom, in linear and logarithmic scales, respectively. The red model in **a** is the same as those in Fig. 6 **b** and Fig. S3 **a**, except the x-length (from –500 to 500 μm). The z-distance of the intercellular and return current is 200 μm in each circuit. Continuous lines represent the propagating intercellular current, while dotted lines are the return current. The blue model is composed of eight circuits with a 1 μA current conducted in each circuit. In b, note that the magnetic field of the computer model (red) used similar to that in Fig. 6 **b** declines to 30 fT at 15 mm below the origin *O*. In the second model (blue) with an intercellular current symmetrically surrounded by return currents, the magnetic field declines more rapidly with distance. When the intercellular current position is shifted from the centre (z = 0) to –150 μm, the decrease in the magnetic field is slowed similar to the red model circuit: 21 fT at 15 mm (data not shown).
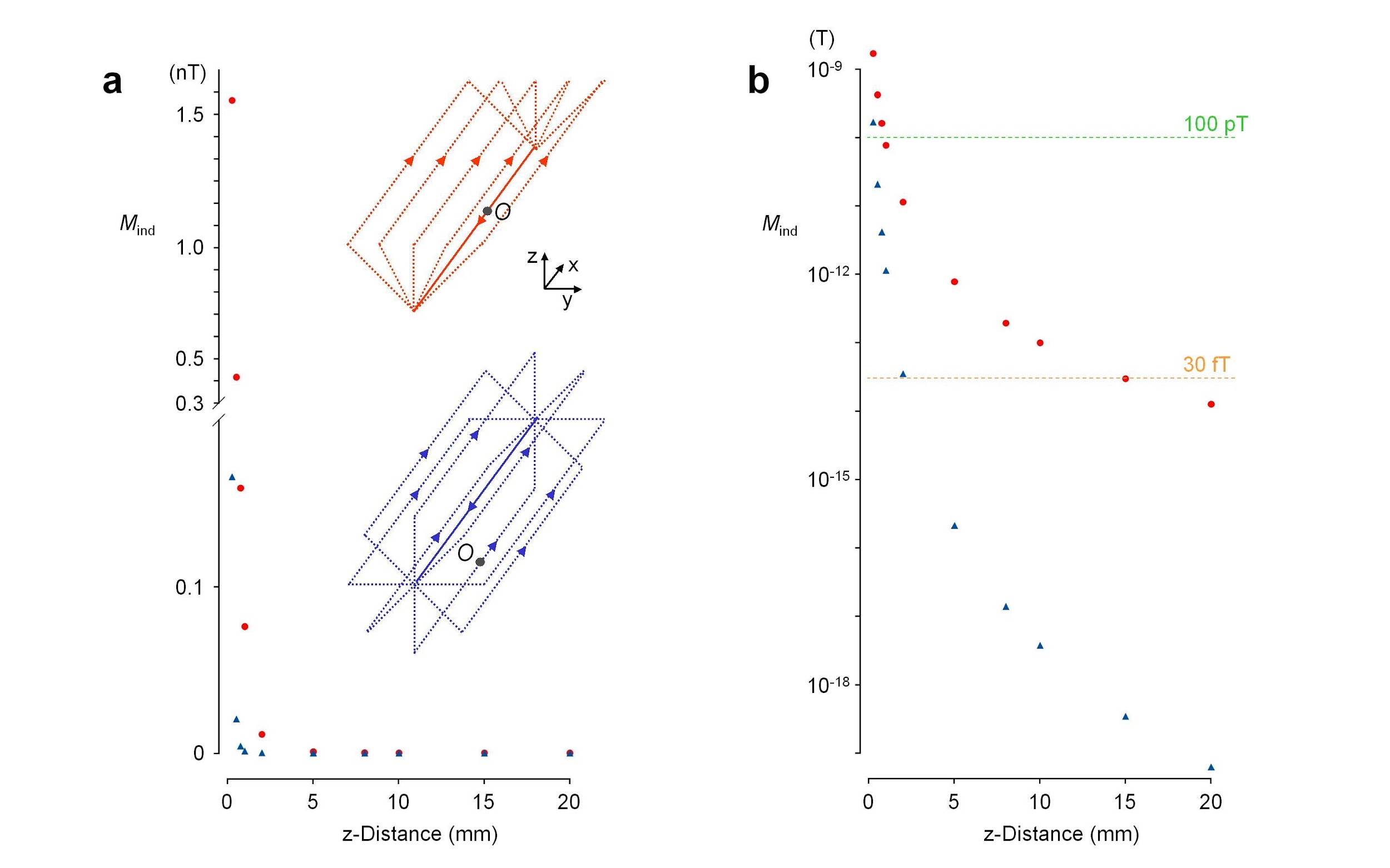


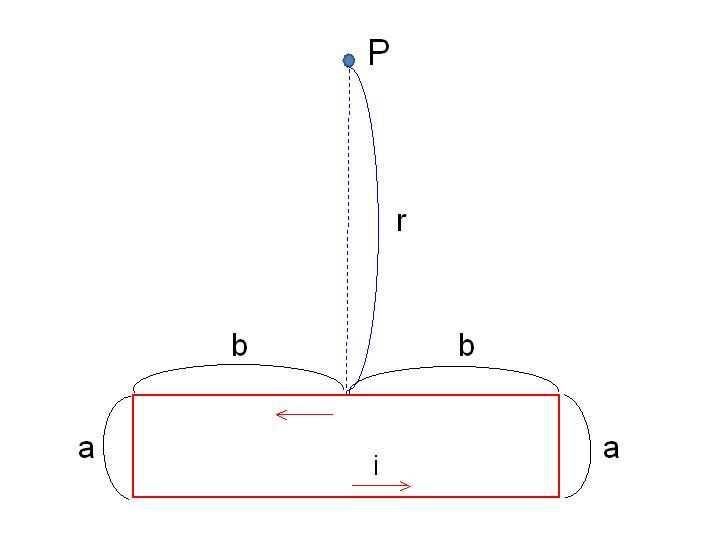


**Appendix |** **A simple model circuit for estimating biomagnetic fields.** To understand how the distance between intercellular propagating current and extracellular return current affect measurement of biomagnetic fields, the model circuit in Fig. 6 is simplified as a rectangular circuit with the current segments (a) and (2b). The two segments (2b) represent the intercellular propagating current in cellular organization and the accompanying extracellular return current, respectively. The length of the segment (a) corresponds to the distance between intercellular propagating current and extracellular return current (i.e. thickness of a bioelectric circuit). The segment (r) represents the distance between magneto sensor and bioelectric circuit. The sensor is placed at (P) in the center of the current segment (2b).

The intensity of the magnetic field at P (H_P_) is described as the sum of the intensity of magnetic fields:

H_P_ = H_iv_ + H_ip_ , (Eq1)

where H_iv_ and H_ip_ are of the magnetic fields induced by electric currents vertical to and parallel with the segment (r), (corresponding to (2b) and (a) current segments), respectively. Note that magnetic fields are expressed as magnetic flux density (B) in this paper. So, the equations of magnetic field density (H) in this section need to be converted with the magnetic permeability in space (i.e. B = μ_0_H)

The intensity of the magnetic field induced by each current segment of the rectangular circuit above is estimated by using the following formula:

H = (i /4πr) (cosθ_1_ + cosθ_2_) , (Eq2)

where (i) represents the amplitude of the linear electric current segment (AB), and (r) represents the distance between the observation point (P) and the current segment. θ_1_ and θ_2_ are the angles of (A) and (B). The direction toward the reverse side from the right side of this paper is defined as positive in the magnetic field of the equation.
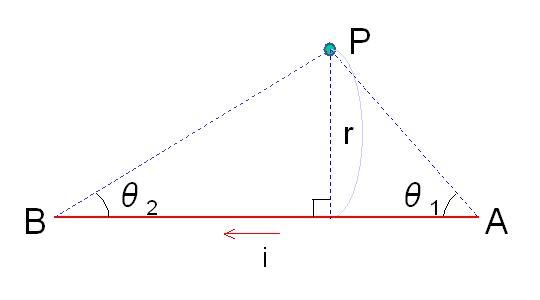


In the simple bioelectric circuit, since the line segment (r) intercepts the segment (2b) at the center, cosθ_1_ equals cosθ_2_. Thus, H_iv_ and H_ip_ are expressed as a function of (a), (b) and (r):

H_iv_ = (b i /2π) [1 / {r √(r^2^ + b^2^)} - 1 / {(r + a) √((r + a)^2^ + b^2^)}] , (Eq3)

H_ip_ = (- i /2πb) [(r + a) / {√((r + a)^2^ + b^2^)} - r / {√(r^2^ + b^2^)}] , (Eq4)

When (r) » (a), (r + a) ≈ (r): This corresponds to a condition of measurement in which the magneto sensor used is largely separated from the bioelectric circuit in the cellular organizations, despite a small distance between the intercellular and extracellular currents. Under this condition, the magnetic fields H_iv_ and H_ip_ become naught. Thus, in order to measure sufficient magnetic field, it is necessary to keep the sensor with the distance (r) comparable to (a).

When the sensor is mounted in a position where (r) and (a) are comparable, and when the distance of bioelectric current propagation (b) is sufficiently longer than both (r) and (a), the inside of the root thus becomes unit in the following equations derived from Eq3 and Eq4 by cancellations with (1/b):

H_iv_ = (b i /2π) [(1/b) / {r √((r/b)^2^ + 1)} - (1/b) / {(r + a) √(((r + a) / b)^2^ + 1)}] , (Eq5)

H_ip_ = (-i /2πb) [(1/b) (r + a) / {√(((r + a) / b)^2^ + 1)} - (1/b) r / {√((r/b)^2^ + 1)}] . (Eq6)

With (r/b) and ((r + a)/ b) ≈ 0, these equations are simplified as:

H_iv_ = (i /2π) [1 / r - 1 / (r + a) ] = ( i /2π) [a / {r (r + a)}] , (Eq7)

H_ip_ = (-i /2πb) [(1/b) (r + a) - (1/b) r] = (-i a / 2πb^2^). (Eq8)

In Eq8, when (b) » (a), H_ip_ becomes naught, unless (i) » (b): Practically, the amplitude of the electric current (i) (in μA) is considered to be smaller than the length of (b) (in mm). This means that the contribution of parallel current is negligible, when the distance of electric current propagation is sufficiently long. Also, Eq7 indicates that the local magnetic field H_P_ becomes nearly identical to H_iv_ (H_P_ ≈ H_iv_), which is independent of the length (b), and is expressed as a function of the sensor distance (r) and circuit thickness (a). This condition corresponds to the experiments in Fig. 5.

When Eq7 is further simplified by assuming (a) » (r), (a) / (r + a ) ≈ 1. Thus,

H_P_ = H_iv_ = ( i /2πr) . (Eq9)

This equation is identical to Ampere’s circuital law, and also corresponds to the experimental condition in Fig. 2.
